# Dynamic splinting home therapy for toe walking: a case report

**DOI:** 10.1186/1757-1626-2-188

**Published:** 2009-11-10

**Authors:** Pamela Lundequam, F Buck Willis

**Affiliations:** 1 Methodist Hospital, Park Nicollet Rehabilitation Services Park Nicollet Blvd, St Louis Park, MN 55416, USA; 2 University of Phoenix, Axia College, E. Elwood St., Phoenix, AZ 85034, USA; 3 Dynasplint Systems, Clinical Research, Ritchie Highway, Severna Park MD 21146, USA

## Abstract

Serial casting is frequently prescribed for toe-walking but that does not allow continued physical therapy (PT). This report described a child and family who chose dynamic splinting (DS) with concurrent PT for treatment. The patient presented with right hemiparesis; below average motor skills and a gait pattern of toe contact (without ankle foot orthosis). Four months of PT plus 6 hours/night of DS as home therapy, the patient's passive dorsiflexion increased 14° and she gained the ability to walk in "flat foot" contact without the Ankle Foot Orthosis. This concurrent treatment achieved improved gait pattern and strength training not possible with casting.

## Introduction

Toe walking is a condition that impairs the most common Activity of Daily Living (ADL), ambulation. A substantial number of patients suffer from this pathology, which may be due to underlying neurological disease (i.e. cerebral palsy) or may be idiopathic in nature [1-6]. Excessive ankle plantarflexion affects a child's gait pattern and disables the ability for functional play, such as single leg standing, jumping or hopping, and all symmetrical bilateral coordination activities [4,7]. Treatment of toe walking impairment includes managing any neuromuscular tone and reducing any contracture, including the molecular shortening of the connective tissue.

Current "Standard of Care" for toe walking includes Botulinum Toxin-A (Botox) injections for tone management [1,3,6] and physical therapy following serial casting for contracture reduction [4,5]. The Botox injections to the gastrocnemius-soleus complex have proven to be an effective short term method by blocking the acetylcholine receptors to disrupt the neuromuscular junction. Botox alone lasts three to four months and is effective for short terms in tone management but has no effect on contracture reduction [6]. Contracture reduction is accomplished by manual therapy and serial casting for six to twelve weeks [1]. Serial casting deters ADLs and may only be effective for a limited period after the casting is removed [4]. The study by Brower et al. showed that while serial casting was effective for increasing dorsiflexion, 25% of the cerebral palsy patients resumed a symptomatic gait pattern, six weeks after the casting was removed [4].

Altered gait pattern with excessive plantarflexion is the chief complaint of this pathology. Therefore, contracture reduction must be maintained. If Botox, serial casting, and physical therapy are effective, then a fourth intervention is still required to maintain the range of motion (ROM). Often, an Ankle-foot orthoses (AFO) is used to maintain the flexibility that has been gained with previous treatment(s) [3,5]. While the AFO is effective in maintaining ROM, it does not work to increase ROM. The AFO holds the child's foot in the neutral position for ambulation which may allow decreases in the end range as previously gained by serial casting. When the interventions of Botox, serial casting, and physical therapy do not achieve desired goals, these patients may receive surgical intervention, which includes tenomuscular lengthening of the Achilles tendon. This intervention achieves lengthening, but it impairs the child's full function ambulation by reducing the power in ankle plantarflexion [7,8]. The other option is for the child to ambulate with an inefficient pattern of toe walking.

Dynamic splinting is a modality which assists in contracture reduction and tone management through low-load, prolonged-duration stretch. The modality allows for greater time at each patient's end range of motion, and has been proven effective in reducing contractures in several other joints [9,10]. The purpose of this case report was to reveal the benefit of dynamic splinting in restoring dorsiflexion and functional ambulation in a 5 year old female patient with hemiparesis.

## Case Presentation

### Initial Assessment

A five year old, Caucasian, American girl with right hemiparesis presented at initial evaluation with below average gross motor skills and increased plantar flexion tone in the right lower extremity. Her typical gait pattern without shoes had toe contact on the right and heel strike on the left. At times she would compensate by walking on her toes bilaterally. She wore an articulated AFO on the right which allowed for flat foot contact with gait. She needed one hand assist to hop on the right foot. Single leg balance on the right leg was for 3 seconds. She participated in gymnastics and swimming. The patient was passively stretched as part of a home exercise program (HEP), however gains in Passive Range Of Motion (PROM) were difficult to maintain throughout the day.

Treatment sessions were scheduled every other week to accommodate the patient/family's school/work schedule. Sessions were 50-70 minutes in length and included stretching, manual therapy and active therapeutic exercise to assist dorsiflexion and strength on the right lower extremity. Manual therapy included myofascial release to the right gastrocnemius and posterior talar glides to assist passive dorsiflexion. Stretching was done in a runner's lunge position using manual or positioning tools to achieve near sub-talar with a 30 second hold, repeated 3-4 times. Strengthening activities included: active resisted dorsiflexion with a yellow theraband, 8-10 repetitions; heel walking × 30 feet 2-3 repetitions; toe scrunches with a towel or play-doh; and active dorsiflexion with ball kicks. In addition the patient performed active, therapeutic activities including squats on various surfaces, single leg hops, double leg hops, single leg balance, skipping, and descending stairs. All treatment activities were completed with patient receiving tactile and verbal cues for maintaining appropriate foot/ankle alignment. A HEP was updated regularly and reviewed with parents at every visit.

### Adjunct Intervention

The attending pediatric physiatrist administered Botox injections to the gastrocnemius-soleus complex and suggested serial casting. The physical therapist and the patient's parents decided to use the Ankle Dorsiflexion Dynasplint, a Dynamic Splint (AFD) which would allow the child to continue physical therapy while using this modality at home, as her Home Exercise Program (HEP) to achieve the low-load, prolonged-duration stretch required to reduce contracture.

**Figure 1:**
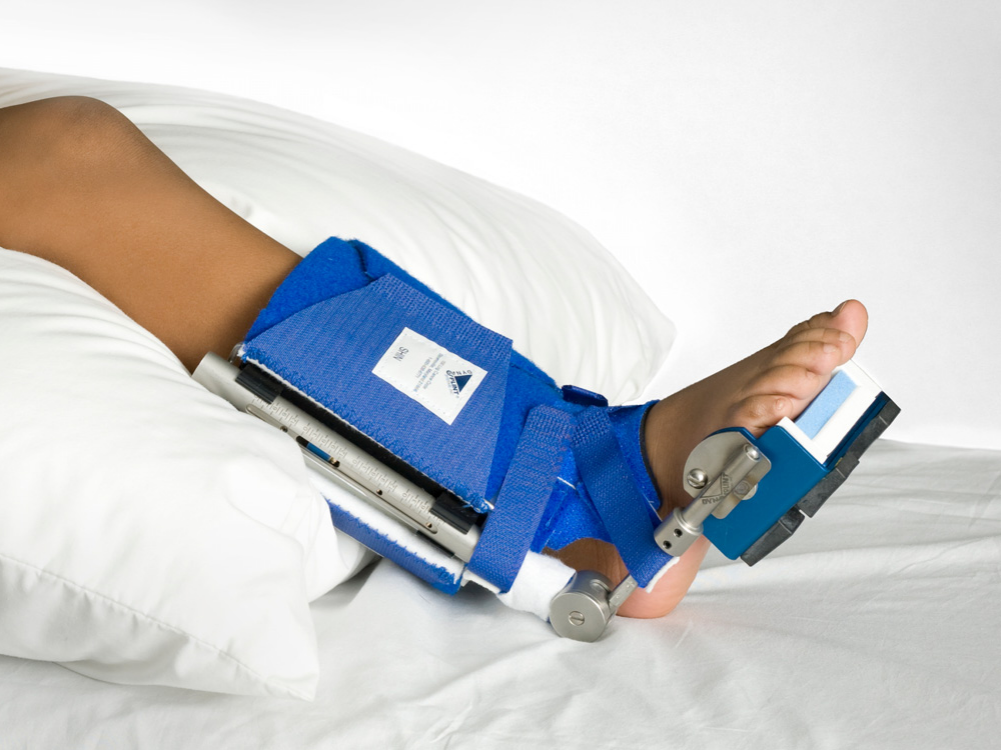
**Ankle Dorsiflexion Dynasplint**. Photograph showing the ankle dorsiflexion dynasplint.

The patient was fit with the AFD (See figure 1) which included customized adjustments to the patient's foot size and leg length. The patient wore the AFD at rest, while wearing the AFO to support her foot/ankle into a neutral position. The patient and her parents were instructed on donning, wear and care, and the protocol for use of this splint. They were instructed to start wearing the AFD for a few hours before bed time on the first day.

After the first day of accommodation, the patient/family was instructed to wear the AFD while she slept, and the tension of the dynamic splint was initially set at tension level #4 equaling 4.1 foot pounds of torque. If excess fatigue followed a session (soreness for more than 1 hour in the morning comparable to soreness after one hour of intense physical therapy) the patient was instructed to decrease the tension one half of a setting for the next two nights. The patient/family were encouraged to increase the tension every two weeks if she was comfortable with those gradual changes in torque until reaching tension level #6 (6.0 foot pounds of torque). Patient compliance was tracked with a daily journal, submitted to the attending clinicians weekly.

Following four months of physical therapy and AFD as HEP, She gained 14° in passive dorsiflexion and gained 9° in active dorsiflexion. The most substantial change was noticed in her gait pattern without the AFO. She was able to walk (barefoot) with flat foot contact on the right and continued heel strike on the left. In addition, she tripled her time in single leg stance (3 seconds initially, progressed to 10 seconds) and she gained the ability to single leg hop. All of these gains allowed her to learn to skip with a symmetrical pattern and play more efficiently with her peers.

## Discussion

The benefit of combining Botox for tone management with serial casting for contracture reduction, has been shown[1], but this is the first report to demonstrate the benefit in using dynamic splinting following Botox injections. This modality has been used for knee, ankle, wrist, finger, toe, and elbow contracture reduction for 25 years. It has recently been adapted for other joints such as the shoulder, and to treat conditions such as carpal tunnel syndrome and trismus. The combination of Botox and dynamic splinting allows the therapist to continue with beneficial therapeutic protocols (such as manual therapy) that would have been eliminated while in casts. Dynamic splinting has been shown effective in reducing excessive plantarflexion contracture in both CVA and TBI patients [9].

The purpose of this case report was to reveal the benefit of dynamic splinting in restoring dorsiflexion and functional ambulation in a 5 year old female patient with hemiparesis. Dynamic splinting, used as a home therapy, provided 42-56 hours per week of additional stretching at end range. This was accomplished with controlled, dynamic tension which adapted to gains in ROM, while maintaining the ankle joint at end range.

For therapists, the use of dynamic splinting in conjunction with prescribed physical therapy may prove more beneficial than serial casting because it allows the patient to enjoy many activities that casting would eliminate for 8 to 12 weeks. An additional benefit of the AFD is that skin integrity is not an issue as it often is with serial casting. Therefore, treatment for contracture reduction is continuous which may lead to improved long term results in ambulation. The unit can be worn nightly for several years if required. Using the AFD allows clinicians to combat toe walking in a three pronged, concurrent attack: Botox, Physical Therapy, and Dynasplint as home therapy.

## Conclusion

The patient presented with below average gross motor skills and increased plantar flexion tone in the right lower extremity, resulting in toe-walking when not wearing an articulated AFO. Following four months of physical therapy and an additional 720 hours of stretching at end range (tracked through weekly reports) with the AFD home therapy, She gained 14° in passive dorsiflexion, gained 9° in active dorsiflexion, and returned to flat foot contact in ambulation without the AFO. After this successful treatment, The girl learned to play hopscotch with her friends.

## Patient's Perspective

The patient's parents found the AFD to be a relatively easy way to achieve follow-through with the prolonged passive stretching portion of her HEP. She tolerated the AFD well most nights with waking on occasion and parents would remove the AFD, although the patient would not remember this in the morning. Increased waking during the night was noted to when the effectiveness of her Botox was wearing off. The substantial time in home therapy (720 hours) was more than the family could have ever devoted to clinic visits.

## List of Abbreviations

(AROM): Active Range of Motion; (AFD): Ankle Dorsiflexion Dynasplint; (AFO): Ankle Foot Orthosis; (DS): Dynamic splinting; (HEP): Home Exercise Program; (ROM): Range of motion.

## Competing interests

Dr. Lundequam has no competing interest. Dr Willis is employed by Dynasplint Systems Inc, but he has not ownership or stock options with this company.

## Authors' contributions

PL was responsible for patient recruitment and treatment. PL was also responsible for development and completion of this manuscript. FBW contributed literature review, data analysis, and development of this manuscript. Both authors read and approved the final manuscript.

## Consent

Written informed consent was obtained from the patient (parents) for publication of this case report and accompanying images. A copy of the written consent is available for review by the Editor-in-Chief of this journal.
